# The Biphasic Activity of Auricularia Auricula-Judae Extract on Bone Homeostasis through Inhibition of Osteoclastogenesis and Modulation of Osteogenic Activity

**DOI:** 10.4014/jmb.2408.08055

**Published:** 2024-10-28

**Authors:** Shin-Hye Kim, Hye-Lim Shin, Tae Hyun Son, Dongsoo Kim, Hwan-Gyu Kim, Jae-Han Cho, Sik-Won Choi

**Affiliations:** 1 Forest Biomaterials Research Center, National Institute of Forest Science (NIFoS), Korea Forest Service (KFS), Jinju 52817, Republic of Korea; 2 Department of Biological Sciences, Jeonbuk National University, Jeonju 54896, Republic of Korea; 3 Postharvest Research Division, National Institute of Horticultural and Herbal Science (NIHHS), Rural Development Administration (RDA), Wanju 55365, Republic of Korea

**Keywords:** *Auricularia auricula-jadae*, osteoporosis, bone hemostasis, osteoblasts, osteoclasts

## Abstract

Osteoporosis arises from the disturbance of bone homeostasis, a process regulated by osteoblasts and osteoclasts. The treatment and prevention of bone metabolic disorders resulting from an imbalance in bone homeostasis require the use of agents that effectively promote both bone formation and anti-resorptive effects. Therefore, an investigation was carried out to determine the potential of the edible mushroom *Auricularia auricula-judae* in modulating bone remodeling by inhibiting RANKL-induced osteoclastogenesis and enhancing BMP-2-stimulated osteoblast differentiation. Moreover, this study assessed the mode of action of the *Auricularia auricula-judae* extracts. The staining of tartrate-resistant acid phosphatase (TRAP), a marker for osteoclast activity, demonstrated that *Auricularia auricula-judae* water extract (AAJWE) inhibited the formation of multinucleated osteoclasts while exhibiting no cytotoxic effects. The study demonstrated that AAJWE reduced RANKL-induced osteoclast differentiation by inhibiting c-Fos/NFATc1 through the inhibition of ERK and JNK phosphorylation during the RANKL-induced osteoclast differentiation. Moreover, AAJWE exhibited a dose-dependent induction of ALP expression in the presence of BMP-2 during osteoblast differentiation. The AAJWE strengthened BMP-2-induced osteogenesis through the activation of Runx2 and Smad phosphorylation. Therefore, AAJWE emerges as a promising candidate for both prevention and therapy owing to its biphasic effect, which aids in the preservation of bone homeostasis.

## Background

Osteoporosis is a widespread bone disorder that poses a significant public health challenge within the human population. This condition affects a considerable number of individuals across all genders and ethnicities, with its prevalence increasing with advancing age [1]. Osteoporosis results from a disruption in bone metabolism, a process that is intricately regulated by osteoblasts and osteoclasts. Current therapeutic options for osteoporosis include bisphosphonates, Raloxifene, Teriparatide, and vitamin D, among others. However, these treatments are accompanied by specific limitations. For instance, the oral administration of bisphosphonates is often associated with gastrointestinal complications, such as gastroesophageal reflux and erosive esophagitis. Furthermore, many of these treatments can lead to the development of hypocalcemia [2]. Consequently, there is a pressing need to explore novel targets for the early intervention of osteopenia and to develop new pharmacological agents. It is essential to investigate innovative treatment strategies, particularly those aimed at maintaining balanced bone homeostasis in osteoporosis, with a focus on the utilization of natural substances due to their favorable safety profile.

Osteoporosis is a condition that arises from an imbalance between osteoblasts and osteoclasts [3]. Osteoclasts, which are multinucleated cells derived from immune cells of the macrophage lineage, play a crucial role in the resorption of osteocytes [4]. The differentiation of hematopoietic stem cells (monocytes and macrophages) into multinucleated osteoclasts is a complex multistep process including cell differentiation, migration, fusion, and resorption. Mononuclear osteoclasts then fuse with each other to form giant multinucleated osteoclasts that subsequently mediate bone resorption [5]. Specifically, excessive bone resorption by overactivated osteoclasts is involved in several bone loss-associated disorders such as osteoporosis, rheumatoid arthritis, osteosarcoma, and cancer bone metastasis [6]. Therefore, understanding the molecular mechanisms by which the cellular components control the osteoclastogenesis is helpful to develop the therapeutic strategies to overcome bone loss-related disorders.

To effectively manage osteoporosis, it is essential to inhibit the differentiation of osteoclasts while concurrently promoting the differentiation of osteoblasts [3]. Osteoblasts are derived from mesenchymal stem cells found in the bone marrow. Osteoblast differentiation is a pivotal event in bone formation. Differentiation of osteoblasts from mesenchymal progenitor cells contributes to bone formation by promoting the production of extracellular matrix, which supports ossification by closely packed sheets on the bone surface [7]. Osteoblast differentiation is regulated by signaling cascades and several transcriptional factors that promote mineralization and formation of bone. Runt‐related transcription factor 2 (Runx2), a transcription factor, is essential for osteoblast differentiation via its ability to induce the expression of osteoblastic downstream effectors [8]. Accordingly, activation of Runx2 represents a therapeutic strategy for treating osteoporosis with bone defect.

Medicinal chemistry strategies for the treatment of osteoporosis frequently result in adverse side effects. Consequently, there is a growing interest in exploring treatment options that incorporate natural products [9]. Among the diverse array of natural product sources, mushrooms have been employed for both culinary and medicinal applications for centuries. The nutritional and pharmacological significance of edible mushrooms is underscored by their status as a source of various biologically active compounds that promote health [10]. Notably, *Auricularia auricula-judae* (AAJ) ranks as the fourth most cultivated edible mushroom globally [11]. Extracts and polysaccharides derived from AAJ exhibit numerous health benefits, including antioxidant, anti-hyperlipidemic, anti-inflammatory, and anti-cancer properties. Additionally, the polysaccharides from AAJ contribute positively to maintaining a balanced gut microbiome [12]. Mushrooms have been documented to play a role in supporting bone homeostasis [13, 14]. Additionally, the antioxidant and anti-inflammatory properties associated with mushrooms are relevant to the maintenance of bone homeostasis [15-18]. Although prior research has emphasized the beneficial effects of *Auricularia auricula-judae* (AAJ) on various health parameters, the specific influence of *Auricularia auricula-judae* extract (AAJE) on bone health remains largely unexplored. Given the established correlation between antioxidant and anti-inflammatory properties and bone homeostasis, it is reasonable to hypothesize that AAJE, which possesses recognized antioxidant and anti-inflammatory activities, may exert a positive effect on bone health. However, the effects of AAJE on bone homeostasis have not been previously examined. Therefore, this study seeks to investigate bone homeostasis as a novel therapeutic approach for addressing bone-related disorders, utilizing extracts derived from *Auricularia auricula-judae*.

## Methods

### Preparation of AAJ Extracts

AAJ was cultivated by and collected from the National Institute of Horticultural and Herbal Science (Rural Development Administration, Republic of Korea) in 2020. It was washed with clean sterile water and air-dried at 50°C for three days to remove moisture. Subsequently, the dried AAJs powder (100 g) were subjected to defatting with hexane (1 L) three times over a period of 24 hs at room temperature. Following this process, the mixture was filtered using Whatman No. 2 filter paper. The residual AAJs were then extracted three additional times with fermented ethanoldms 1 L, (Daejung chemicals & metals Co. Ltd., Republic of Korea), with filtration performed through a Büchner funnel lined with Carl Roth filter paper (111A, Ø100 mm, Karlsruhe, Germany). The resulting filtrates were combined and concentrated using a rotary evaporator. The residues obtained from the ethanol extraction underwent two further extractions with water (1 L) for 24 h at room temperature. Afterward, the extracts were combined and dried using a freeze dryer. All extracts were subsequently solubilized in dimethyl sulfoxide (DMSO). The control group was treated with DMSO alone.

### Reagents

Recombinant human BMP-2 (rhBMP-2), mouse soluble RANKL, and M-CSF were purchased from R&D Systems (USA) and reconstituted in 0.1% bovine serum albumin in Dulbecco’s phosphate-buffered saline (DPBS) according to the manufacturer’s specifications. DPBS was purchased from Cytiva (USA). Dimethyl sulfoxide (DMSO) was procured from Sigma Aldrich (USA), and the vehicle group exclusively employed DMSO. TRIzol was purchased from Invitrogen (USA). Alpha-minimum essential medium (α-MEM) for cell culture, fetal bovine serum US origin (FBS), and antibiotics (penicillin and streptomycin) were purchased from Gibco (Thermo Fisher Scientific, USA). Antibody against ALP were purchased from R&D Systems. Antibodies against NFATC1, c-Fos, Smad, and Actin, as well as horseradish peroxidase (HRP)-conjugated anti-mouse and anti-rabbit antibodies, were purchased from Santa Cruz Biotechnology (USA). Antibodies against p-ERK, ERK, p-JNK, JNK, p-p38, p38, p-AKT, AKT, Runx2, p-Smad were purchased from Cell Signaling Technology (USA).

### Ethics Statement

This study was performed using the Standard Protocol's suggested procedures for Animal Studies of the Department of Laboratory Animal Resources, Yonsei Hospital Biomedical Research institute. The experimental protocol was approved by the Institutional Animal Care and Use Committee of Yonsei Hospital Biomedical Research institute (Permit no. 2022-0104). All efforts in this study were made to minimize suffering, stress/discomfort, and the number of animals.

### Osteoclast Differentiation

All experiments were conducted in accordance with previously established protocols, with certain modifications implemented [14]. Osteoclast differentiation was performed as previously described [19]. Primary bone marrow-derived macrophages (BMMs) for osteoclast differentiation were gotten as follows. We selected five-week-old male mice from the Institute of Cancer Research (ICR; ORIENT BIO., Republic of Korea). The breeding room conditions were set to a 12-h light on/off cycle, controlled temperature (22–24°C), and a humidity of 50–60%. The mice were provided standard food and water ad libitum. To obtain BMMs from the ICR mice, we flushed the femur and tibia of the leg with α-MEM supplemented with antibiotics. The bone marrow cells were spread on a 10-cm cell culture dish containing α-MEM supplemented with 10% FBS and M-CSF (10 ng/ml) and incubated for 1 d. Cells that did not adhere to the culture dishes were transferred to fresh culture dishes. The cells were grown in M-CSF (30 ng/ml) supplemented α-MEM for 3 days. After washing away the unattached cells, the cells attached to the culture dish were harvested and used as BMMs. To differentiate BMMs into osteoclasts, the former was seeded at a density of 1.5 × 10^5^ cells/ml in a 6-well plate or 10^5^ cells/ml in a 96-well plate and incubated with M-CSF (50 ng/ml) and RANKL (30 ng/ml) for 4 d. All media were changed every 3 d. Multinucleated osteoclasts were observed under an inverted microscope.

### Tartrate-Resistant Acid Phosphatase Staining and Activity Assay

Differentiated osteoclasts were confirmed by staining with tartrate-resistant acid phosphatase (TRAP), a marker of osteoclast differentiation. Mature osteoclasts were fixed in 10% formalin for 5 min, rinsed with distilled water, and then stained using a TRAP Staining Kit (USA). TRAP-expression cells bear at least three nuclei; therefore, TRAP-positive multinucleated osteoclasts (nuclei ≥ 3) were counted using an inverted microscope. To measure TRAP activity, a marker of mature osteoclasts, multinucleated osteoclasts were fixed in 10% formalin for 5 min, rinsed with distilled water, and treated with TRAP buffer (100 mM sodium citrate, pH 5.0, 50 mM sodium tartrate) supplemented with 3 mM p-nitrophenyl phosphate (PNPP; Sigma-Aldrich), the substrate of TRAP, at room temperature for 20 min. To stop the reaction, the sample including the TRAP buffer was placed in a different plate with 50 μl of 0.1 N NaOH as the stop solution. The optical density of the solution was then determined at 405 nm using a spectrophotometer (SpectraMax iD3, Molecular Devices, USA).

### Osteoblast Differentiation

Obtained from the American Type Culture Collection (USA), C2C12 mouse mesenchymal precursor cells were used for osteoblast differentiation. C2C12 cells were cultured in α-MEM supplemented with 10% FBS and antibiotics without ascorbic acid. The cells were incubated at 37°C in a 5% CO_2_ atmosphere incubator (; MCO-170AIC-PK, Panasonic, Japan), and their medium was changed every 2–3 d. For osteoblast differentiation, C2C12 cells were plated on 96-well plates at 2.5 × 10^4^ cells/ml or in 6-well plates at 1.25 × 10^5^ cells/ml and maintained in α-MEM containing 10% FBS and antibiotics. After culturing for 1 d, the medium was changed to α-MEM supplemented with 5% FBS and rhBMP-2 (50 ng/ml) for osteoblast differentiation. Osteoblast differentiation was monitored by ALP staining.

### ALP Staining and Activity Assays

Osteoblastic differentiation of C2C12 cells was assessed by ALP staining and activity. C2C12 cells were differentiated for 3 d under osteogenic conditions (BMP-2 stimulation) in the presence of vehicle or AAJE. Subsequently, the differentiated cells were washed with DPBS and fixed with 3.7% formaldehyde for 5 min, rinsed with distilled water, and stained with BCIP^®^/NBT Liquid Substrate System (Sigma-Aldrich). To determine ALP activity, BMP-2-treated cells were fixed with 3.7% formaldehyde for 4 min, washed with dis-tilled water, and then treated with 1-Step PNPP Substrate Solution (Thermo Fisher Scientific) at room temperature for 20 min. To stop the reaction, the PNPP solution was transferred to other plates containing 50 μl of 0.1 N NaOH as stop solution, and the optical density of the solution was measured at 405 nm using SpectraMax iD3.

### Cell Cytotoxicity Assay

The Cell Counting Kit 8 (CCK-8) assay (Dojindo Molecular Technologies, USA) was performed to examine the cytotoxic effects of AAJE on BMMs and C2C12 cells. The cells were seeded in a 96-well plate and cultured for 3 d with various concentrations of AAJE. The cells were then transferred to media containing the CCK-8 solution for 30 min. The optical density was determined at 450 nm using SpectraMax iD3.

### RNA Extraction and Quantitative Reverse-Transcription PCR Analysis

Primer pairs were generated using online Primer3 software [20]. Table 1 summarizes the primer pairs used in this study. Briefly, total RNA from BMMs and C2C12 cells was extracted using the TRIzol reagent according to the manufacturer’s protocol. cDNA was prepared from 1 μg of total RNA using the RevertAid First Strand cDNA Synthesis Kit (Thermo Fisher Scientific) according to the manufacturer’s protocol for quantitative reverse-transcription PCR (qRT-PCR). SYBR green-based qRT-PCR was performed using the QuantStudio 5 Real-Time PCR System (Thermo Fisher Scientific) and PowerUp SYBR Green Master Mix (Thermo Fisher Scientific). All reactions were performed in triplicate, and the data were analysed using the 2^–ΔΔCT^ method as described by Livak and Schmittgen [21]. Glyceraldehyde 3-phosphate dehydrogenase (GAPDH) or hypoxanthine phosphoribosyltransferase 1 (HPRT1) were used as internal controls.

### Western Blot Analysis

The treated cells were lysed in radioimmunoprecipitation assay lysis buffer (Cell Signaling Technology), containing protease inhibitors. After centrifugation at 15,000 g for 15 min, the protein content in the supernatant was measured using a detergent-compatible protein assay kit (Bio-Rad, USA). Proteins were subjected to sodium dodecyl sulphate-polyacrylamide gel electrophoresis and transferred to polyvinylidene difluoride membranes (Merck Millipore, Germany). The membranes were then incubated with the relevant primary and HRP-conjugated secondary antibodies. The reaction was developed using Clarity Western enhanced chemiluminescence substrate (Bio-Rad) and visualized with ChemiDoc XRS+ (Bio-Rad). β-Actin was used as an internal control.

### Statistical Analysis

**Fig. 1:**
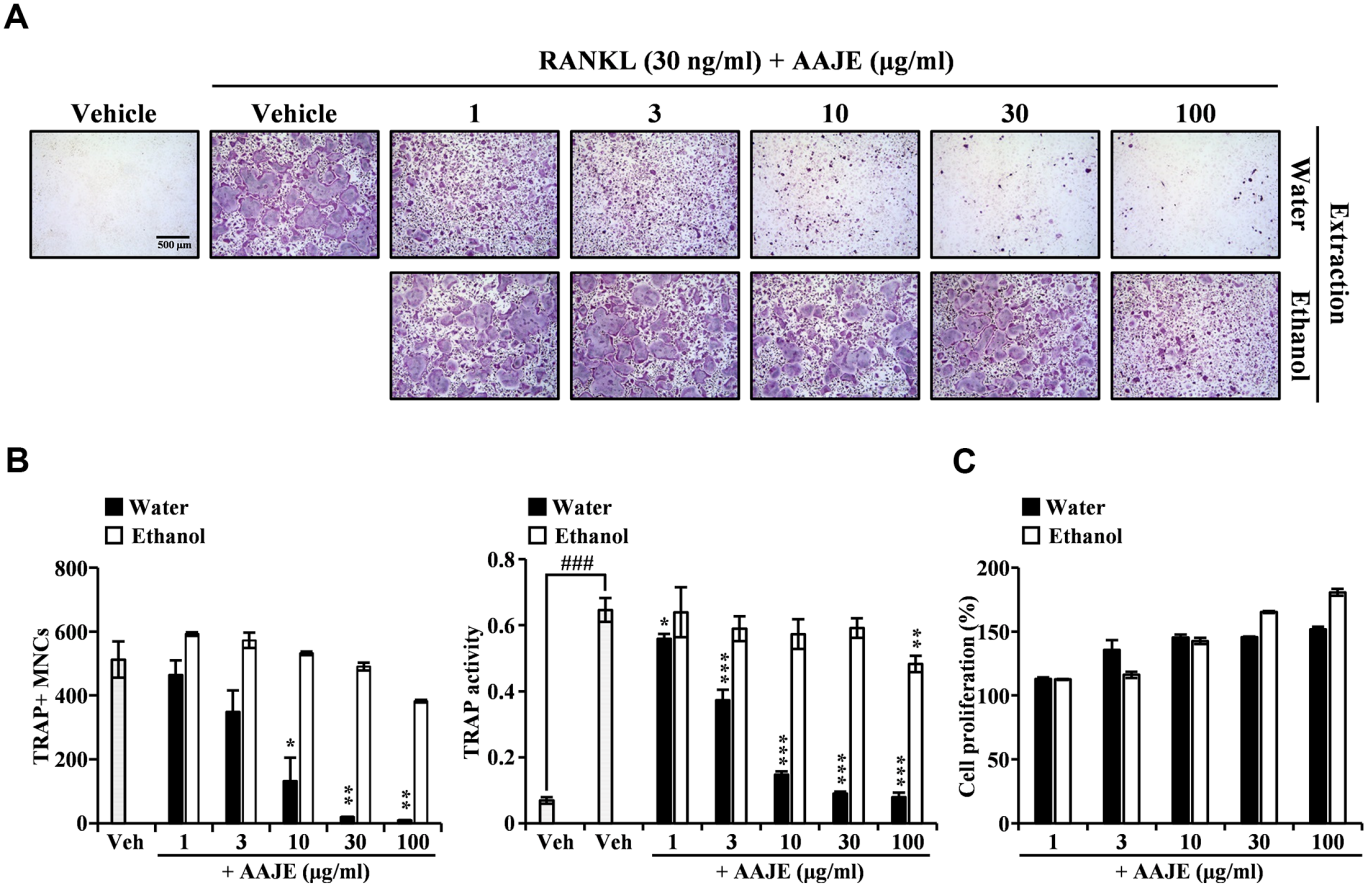
AAJWE Inhibits RANKL-Induced Osteoclastogenesis in BMM Cells. (**A**) Pre-osteoclast cells were cultured with either the vehicle (DMSO) or AAJE at concentrations of 1, 3, 10, 30, and 100 μg/ml, concurrently with M-CSF (50 ng/ml) and RANKL (30 ng/ml) for a duration of four days. Following this incubation period, multinucleated osteoclasts were visualized using tartrate-resistant acid phosphatase (TRAP) staining. (**B**) The number of TRAP-positive multinucleated osteoclasts was quantified (left panel), and TRAP activity was assessed (right panel). Statistical significance was determined with *p < 0.05; **p < 0.01; ***p < 0.001, compared to the vehicle control. (**C**) The effect of AAJE on the viability of pre-osteoclast cells was evaluated using the CCK-8 assay, conducted in triplicate.

**Fig. 2:**
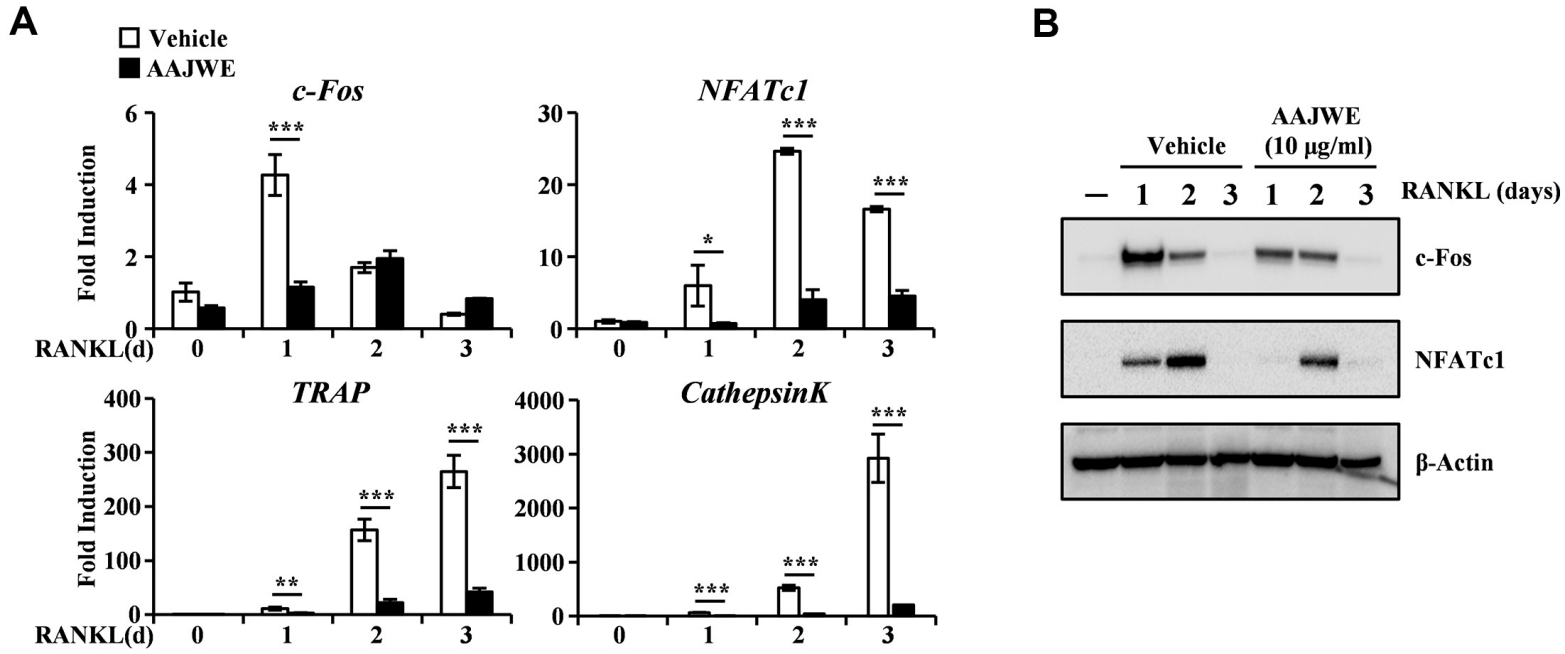
The inhibitory effect of AAJWE on RANKL-induced expression of transcription factors, including c- Fos and NFATc1. (**A**) Bone marrow-derived macrophage (BMM) cells were stimulated with M-CSF (50 ng/ml) and RANKL (30 ng/ml) in the presence of either a vehicle control (DMSO) or AAJWE (10 μg/ml) for the specified duration. Total RNA was subsequently extracted using TRIzol reagent, and mRNA expression levels were assessed via real-time PCR, with GAPDH serving as the internal control. Statistical significance was determined with *p < 0.05; **p < 0.01; ***p < 0.001, compared to the vehicle control. (**B**) The impact of AAJWE on the protein expression levels of RANKL-induced transcription factors was analyzed through Western blotting. BMM cells were pre-treated with either the vehicle or AAJWE (10 μg/ml) prior to stimulation with RANKL (30 ng/ml) and M-CSF (50 ng/ml) for the indicated duration. Cell lysates were subjected to SDSPAGE and transferred to a PVDF membrane. Western blotting was conducted using specific antibodies for each molecule as indicated, with β-Actin utilized as the internal control. A representative result from three independent experiments, which yielded consistent findings, is presented.

**Fig. 3:**
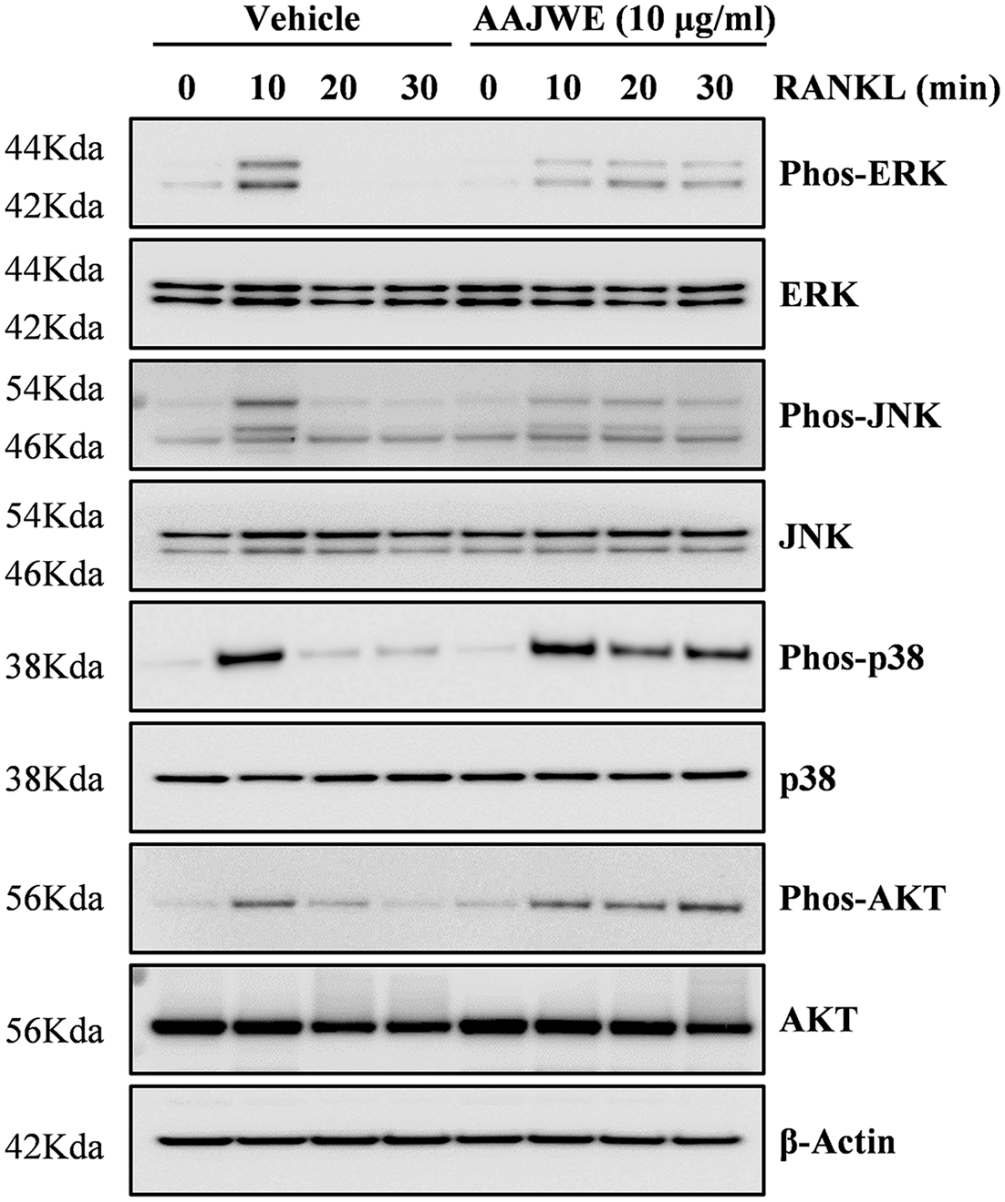
AAJWE modulates RANKL-mediated phosphorylation of ERK and JNK. The expression levels of molecules associated with osteoclast differentiation signaling were assessed using Western blot analysis. Following a 1-day serum starvation period, bone marrow-derived macrophage (BMM) cells were pre-treated with either a vehicle or AAJWE (10 μg/ml) for 1 h prior to stimulation with RANKL (30 ng/ml) for the specified durations. β-Actin served as the internal control. A representative result from three independent experiments which yielded consistent findings is presented.

**Fig. 4:**
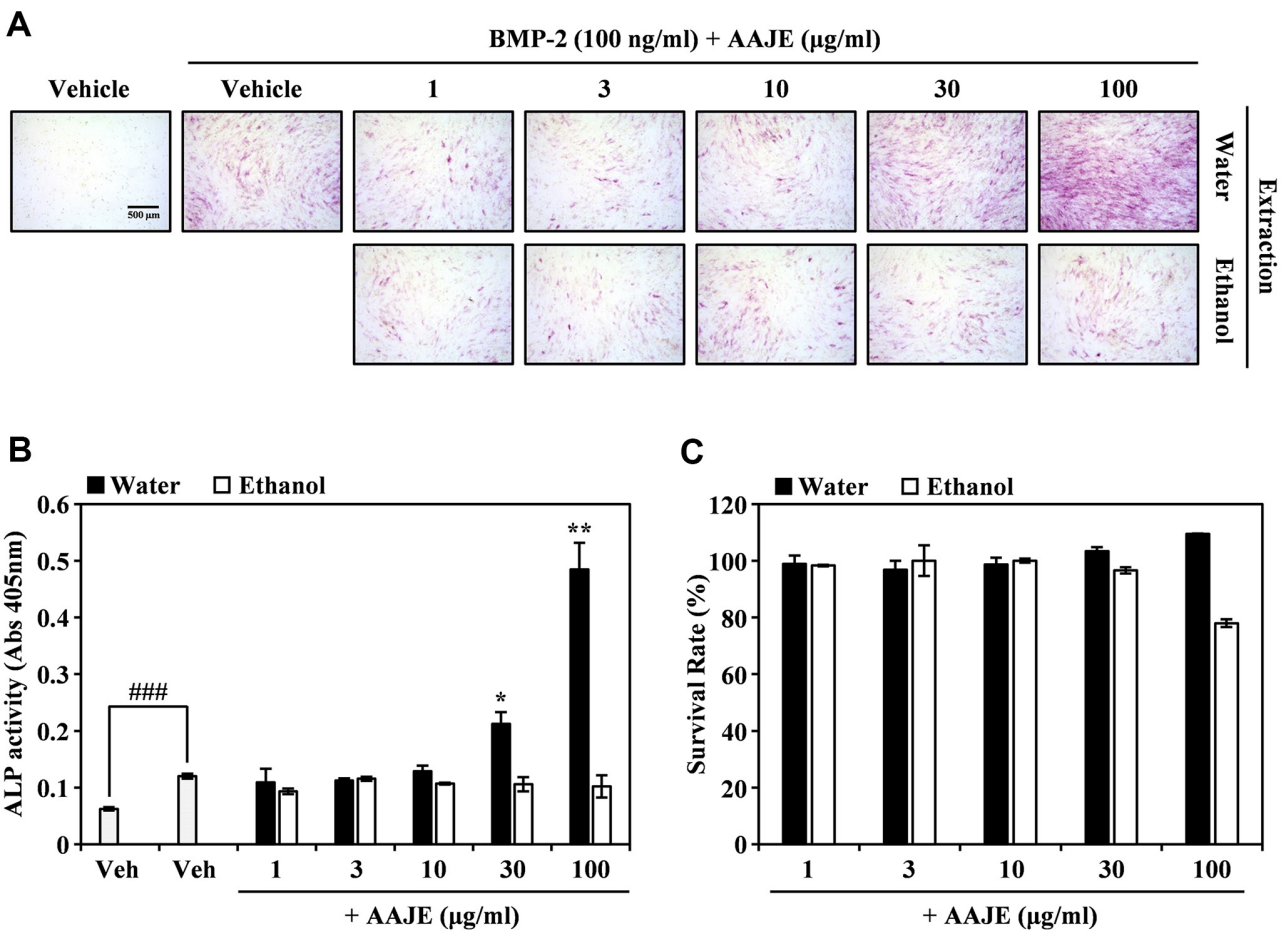
AAJWE enhances BMP-2-induced ALP activity associated with bone formation in C2C12 cells. (**A**) C2C12 cells, a mouse skeletal muscle cell line, were cultured with either a vehicle control (DMSO) or various concentrations of AAJE (1, 3, 10, 30, 100 μg/ml) concurrently with BMP-2 (50 ng/ml) for a duration of four days. Following incubation, osteoblast differentiation was assessed through alkaline phosphatase staining. (**B**) ALP activity was quantified by measuring absorbance at 405 nm. Statistical significance was determined with *p < 0.05; **p < 0.01 (compared to the BMP-2-treated group); ^###^*p* < 0.001 (compared to the control group). (**C**) The effect of AAJE on the viability of C2C12 cells was evaluated using the CCK-8 assay, conducted in triplicate.

**Fig. 5:**
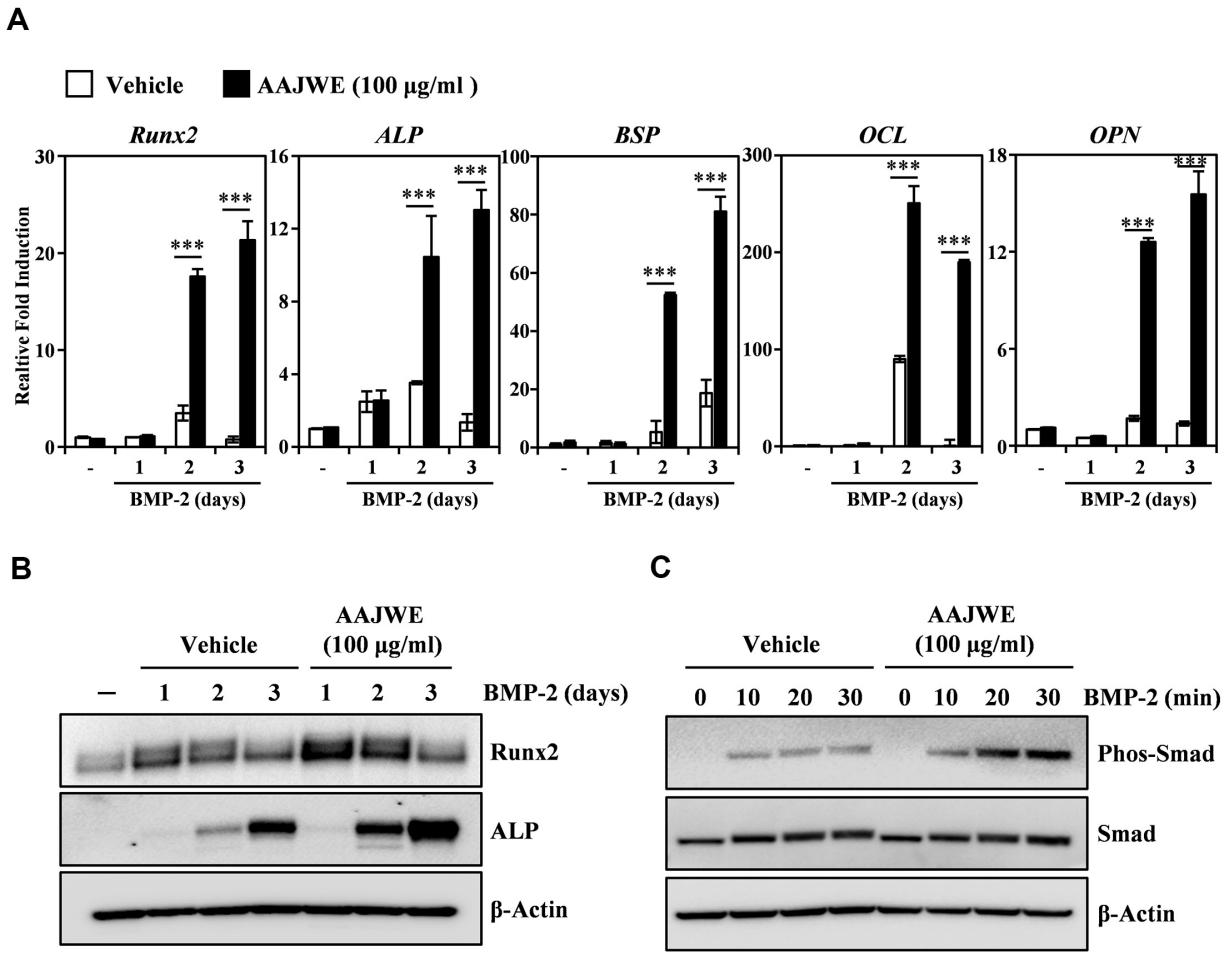
AAJWE enhances BMP-2-stimulated transcriptional activity of Runx2 through the induction of Smad phosphorylation. (**A**) C2C12 cells were stimulated with BMP-2 (50 ng/ml) in the presence of either a control vehicle (DMSO) or AAJWE (100 μg/ml) for the specified durations. The mRNA expression levels were quantified using real-time PCR, with HPRT1 serving as an internal control. Each sample was analyzed in triplicate. Statistical significance was determined with ***p < 0.001 when compared to the vehicle control. (**B**) The effects of AAJWE on the expression levels of Runx2 and ALP were assessed through western blot analysis, utilizing β-Actin as an internal control. (**C**) AAJWE promotes BMP-2-mediated phosphorylation of Smad signaling molecules. Following a 24-h serum starvation period, C2C12 cells were pre-treated with either the control vehicle or AAJWE (100 μg/ml) for 1 h prior to BMP-2 stimulation (50 ng/ml) for the indicated durations. The expression levels of the signaling molecules were evaluated via Western blotting, with β-Actin serving as an internal control. One representative result from three independent experiments, which yielded consistent findings, is presented.

All numerical values are expressed as the mean ± standard deviation. Each experiment included three replicates for each experimental factor and was repeated 3–5 times. Figs. 1–5 show the results of representative experiments. Statistical differences were analyzed using Student’s *t*-test, and significance was set at *p* < 0.05.

## Results

### AAJWE Strongly Inhibited RANKL-Induced Osteoclastogenesis in BMM Cells

To investigate the inhibitory effects of the AAJE fractions on osteoclastogenesis, RANKL-induced BMMs were treated with the AAJE fractions at varying concentrations. As shown in Fig. 1A, both the water and ethanol extracts of AAJ attenuated TRAP-positive osteoclast formation in a dose-dependent manner. TRAP staining showed that AAJWE dramatically inhibited the formation of multinucleated osteoclasts, whereas the ethanol fraction of AAJE showed only a weak inhibitory effect. Consistent with this result, the inhibitory effect of AAJE on osteoclastogenesis was confirmed by counting the number of TRAP-positive multinucleated osteoclasts (TRAP+MNCs; Fig. 1B, left graph) and evaluating TRAP activity (Fig. 1B, right graph). These inhibitory effects were not caused by cytotoxicity, since AAJE did not induce cytotoxicity at concentrations that showed an inhibitory effect in the CCK-8 assay (Fig. 1C). These results suggest that AAJWE significantly inhibited RANKL-induced osteoclast differentiation and its inhibitory effect was greater than that of the ethanol fraction of AAJE.

### AAJWE Attenuated RANKL-Mediated Transcription Factors of Osteoclast-Specific enes

The inhibitory effect of AAJWE on osteoclastogenic activity was investigated by evaluating the expression of osteoclast-specific transcription factors. AAJWE treatment (10 μg/ml) decreased the RANKL-induced mRNA expression of the transcription factors *c-Fos* and *NFATc1*. Furthermore, it inhibited the expression of the osteoclast-related genes *TRAP* and *CathepsinK* at the indicated time points during osteoclast differentiation (Fig. 2A). Western blot analysis confirmed that RANKL-induced upregulation of c-Fos and NFATc1 proteins was inhibited by AAJWE during osteoclast differentiation (Fig. 2B). These results indicated that AAJWE inhibited the osteoclast-specific induction of c-Fos and NFATc1 expression, which is required for osteoclast differentiation.

### AAJWE Inhibited RANKL-Related Activation of ERK and JNK Phosphorylation

To examine the mechanism underlying the anti-osteoclastogenic activity of AAJWE, we investigated its effects on signaling pathways associated with the regulation of c-Fos/NFATc1 triggered by RANKL. We observed that AAJWE treatment attenuated the phosphorylation of ERK and JNK, whereas the activation of p38 and RAC-alpha serine/threonine-protein kinase (AKT) was not affected by the addition of AAJWE (Fig. 3). These findings demonstrated that AAJWE inhibited the early signaling pathways involved in ERK and JNK phosphorylation.

### AAJWE Promotes BMP-2-Stimulated Osteoblast Differentiation in C2C12 Cells

Next, we measured ALP expression to investigate whether AAJE possessed dual activity and could promote BMP-2-induced osteoblast differentiation. Notably, AAJWE stimulated ALP expression in a dose-dependent manner when combined with BMP-2. In contrast, the ethanol extracts of AAJ (AAJEEs) did not demonstrate any promotive effects at the specified concentration, as determined by ALP staining (Fig. 4A). This finding is supported by the observation that AAJWE substantially and dose-dependently increased BMP-induced ALP activity (Fig. 4B). At the cell viability experiment, there was no evidence of cytotoxicity from AAJWE, but AAJEE inhibited C2C12 cell proliferation at 100 μg/ml (Fig. 4C). These results showed that AAJWE synergistically stimulated BMP-2-dependent anabolic activity compared with that of AAJEE alone.

### AAJWE Stimulates BMP-2-Mediated Expression of Transcription Factors Runx2 via Induction of Smad Signaling Pathways

We investigated the osteogenic process of BMP-2-dependent osteoblast development to better understand the anabolic mechanism of AAJWE. The stimulatory effect of AAJWE on osteogenesis was evaluated by measuring the mRNA expression of the osteogenesis-specific transcription factor *Runx2* and its downstream molecules. The addition of AAJWE at a concentration of 100 μg/ml resulted in a synergistic enhancement of *Runx2* mRNA expression. Additionally, the expression of *Runx2*-regulated genes, including *ALP, BSP* (Bone Sialoprotein), *OCL* (Osteocalcin), and *OPN* (Osteopontin), was also upregulated on the specified day during the process of osteoblast differentiation (Fig. 5A). Consistent with this paper previous data, Runx2 and ALP protein levels were also increased by AAJWE addition on the indicated days (Fig. 5B). Furthermore, AAJWE synergistically stimulated BMP-2-induced phosphorylation of Smad at the indicated time points (Fig. 5C). These results suggested that the osteogenic activity of AAJWE during BMP-2-dependent osteoclast differentiation could be involved in the enhancement of the Smad-Runx2 signaling axis for osteogenesis.

## Discussion

This study investigated the effects of *Auricularia auricula-judae* extracts on bone metabolic processes. The results indicate that AAJWE exert a biphasic effect, promoting osteoblast differentiation while concurrently inhibiting osteoclast formation without inducing cytotoxicity. This finding is novel and represents the first documented instance of AAJWE’s role in the regulation of bone homeostasis.

Mushrooms, particularly *Auricularia auricula-judae*, have attracted considerable attention due to their extensively documented health benefits, which encompass a diverse array of biological activities, including antidiabetic, antihypertensive, anti-inflammatory, immunomodulatory, anticancer, antimicrobial, antioxidative, anti-obesity, osteoprotective, and skin wound-healing effects [22-25]. Among these activities, the regulation of bone homeostasis has emerged as a topic of significant interest. This study specifically examined the potential of AAJWE as a promising candidate for interventions aimed at enhancing bone health

Bone homeostasis is a complex equilibrium maintained by the coordinated activities of osteoblasts and osteoclasts. The differentiation of osteoclasts is predominantly influenced by macrophage colony-stimulating factor (M-CSF) and receptor activator of nuclear factor-κB ligand (RANKL) [26]. These cytokines promote the maturation of osteoclast precursors into fully developed, multinucleated osteoclasts, which are responsible for the degradation of bone tissue [27]. This process also initiates the production of enzymes associated with bone resorption, such as tartrate-resistant acid phosphatase (TRAP) and Cathepsin K [26, 28]. In our study, we assessed osteoclast differentiation by quantifying TRAP-positive cells. Our results indicated that AAJWE significantly inhibited osteoclastogenesis without compromising cell viability, whereas ethanol extracts demonstrated comparatively weaker anti-osteoclastogenic effects at elevated concentrations.

To elucidate the underlying mechanisms by which AAJWE mediates the inhibition of osteoclast differentiation, we investigated its effects on transcription factors and signal transduction pathways. RANKL is known to stimulate key transcription factors, specifically c-Fos and NFATc1, which are essential for the differentiation of osteoclasts [29]. The inhibition of c-Fos in osteoclast precursors may also lead to a reduction in osteoclast activity [30]. Recent studies have demonstrated that NFATc1 (-/-) embryonic stem cells are incapable of differentiating into osteoclasts, thereby supporting the assertion that NFATc1 plays a critical role in the regulation of osteoclast differentiation [31]. Additionally, RANKL promotes the activation of the MAPK and NF-κB pathways in both osteoclasts and precursor cells through the transcription factor TRAF6 [32]. During the early phase of osteoclast differentiation, M-CSF and RANKL bind to their respective receptors, activating signaling pathways involving p38, ERK, and JNK [30]. The phosphorylation of MAP kinases may serve as a regulatory mechanism for the transcription factors c-Fos and NFATc1 [33]. Our findings indicate that AAJWE inhibits RANKL-induced osteoclastogenic molecules, including c-Fos, NFATc1, TRAP, and cathepsin K. Furthermore, AAJWE was observed to suppress the phosphorylation of ERK and JNK, which are upstream signaling molecules that modulate the expression of c-Fos and NFATc1. These results suggest that AAJWE inhibits osteoclastogenesis by disrupting the ERK and JNK signaling pathways and downregulating the expression of c-Fos and NFATc1.

Osteoblasts originate from mesenchymal stem cells and are essential for the process of bone formation [34]. Bone morphogenetic protein-2 (BMP-2) serves as a critical regulator of osteoblast differentiation by activating initial signaling molecules, including Runx2 and Smad. These molecules subsequently promote the expression of alkaline phosphatase (ALP), bone sialoprotein (BSP), osteocalcin (OCL), and osteopontin (OPN) [35, 36]. Our study demonstrated that AAJWE significantly increased the number of ALP-positive cells, indicating an enhancement in osteoblast differentiation, while not adversely affecting cell viability.

To further investigate the mechanisms underlying AAJWE-induced osteoblast differentiation, we examined the BMP-mediated signaling pathway. BMP-2 cytokines activate a range of molecular regulators, including osteoblastic transcription factors that are integral to the differentiation of osteoblasts. Runx2 transcription factors play a crucial role in regulating molecules such as alkaline phosphatase (ALP), bone sialoprotein (BSP), osteocalcin (OCL), and osteopontin (OPN) during osteoblast differentiation [37]. ALP is the initial enzyme involved in osteoblast differentiation [38], serving as a significant component of hard tissues and exhibiting high expression levels in mineralized tissues [39]. BSP directly influences osteoblast differentiation by enhancing the production of a reduced matrix [40]. OCL is a marker of mature osteoblasts [41], expressed to a certain degree at the conclusion of osteoblast differentiation and following mineralization [42]. OPN is critical for the mineralization process [43]. The essential role of Runx2 in osteoblast formation has been demonstrated through studies involving knockout mice, ectopic expression of Runx2, and investigations of Runx2 mutations in humans [44-46]. Runx2 haploinsufficiency is linked to cleidocranial dysplasia in humans [47]. Additionally, Runx2 heterozygous knockout mice exhibit osteopenia in adulthood due to reduced bone turnover resulting from impaired osteoblastic function [48]. Conversely, ectopic expression of Runx2 induces endochondral ossification in specific regions of the skeleton [49]. Thus, Runx2 is a critical factor for osteoblast differentiation and the formation of mature bone. In our study, Runx2 demonstrated a synergistic enhancement of mRNA and protein induction following AAJWE treatment in the presence of BMP-2 during osteoblast differentiation. The stimulatory effect of AAJWE on Runx2 induction was corroborated by assessing the transcription of ALP, BSP, OCL, and OPN, which are osteoblast-specific markers. The results indicated that the acceleration of osteoblast differentiation by AAJWE involves the induction of Runx2, a major transcription factor.

BMP-2 activates an osteogenic cellular signaling pathway during the development of osteoblasts through a Smad-dependent mechanism [50, 51]. BMP-regulated Smads, specifically Smad1, Smad5, and potentially Smad8, play a critical role in the differentiation of mesenchymal cells into osteoblasts [52]. The activation of Smad by BMP-2 enhances the expression of Runx2 and Osx, which are integral to osteoblast differentiation [36, 53]. Consequently, we aimed to determine whether the activity of Smad was influenced by treatment with AAJWE. Our findings indicate that AAJWE treatment promotes BMP-induced phosphorylation of Smad. These results suggest that the osteogenic effects of AAJWE during BMP-2-mediated osteoclast differentiation may be associated with the enhancement of the Smad-Runx2 signaling axis, thereby facilitating bone formation.

To the best of our knowledge, this study represents the first report demonstrating that AAJWE exhibits a biphasic activity in the regulation of osteoclast differentiation and osteogenesis, thereby contributing to bone homeostasis. In terms of bone resorption, AAJWE was found to inhibit osteoclast differentiation by blocking the phosphorylation of ERK and JNK, as well as decreasing the expression of transcription factors such as c-Fos and NFATc1. Conversely, in relation to bone formation, our findings indicate that AAJWE is associated with the synergistic induction of the Smad-Runx2 signaling pathways, which are essential for bone formation, including markers such as ALP, BSP, OCL, and OPN. While the study presents compelling evidence, additional research is necessary to comprehensively elucidate the mechanisms underlying the effects of AAJWE. In vivo studies will be crucial to validate its efficacy in animal models of osteoporosis. Furthermore, identifying the specific compounds within AAJWE that contribute to its anti-osteoporotic properties will facilitate the development of targeted therapeutic interventions. Nevertheless, based on the findings, the results presented in this study suggest that the dual action of AAJWE on bone homeostasis positions it as a functional food with potential applications in the treatment and prevention of osteoporosis.

## Figures and Tables

**Table 1:** Primer sequences used in this study.

Target Gene	Forward Primer (5’–3’)	Reverse Primer (5’–3’)
*c-Fos*	CCAGTCAAGAGCATCAGCAA	AAGTAGTGCAGCCCGGAGTA
*NFATc1*	GGGTCAGTGTGACCGAAGAT	GGAAGTCAGAAGTGGGTGGA
*TRAP*	GATGACTTTGCCAGTCAGCA	ACATAGCCCACACCGTTCTC
*Cathepsin K*	GGCCAACTCAAGAAGAAAAC	GTGCTTGCTTCCCTTCTGG
*Runx2*	GACTGTGGTTACCGTCATGGC	ACTTGGTTTTTCATAACAGCGGA
*ALP*	GATGGCGTATGCCTCCTGCA	CGGTGGTGGGCCACAAAAGG
*BSP*	GGCGACACTT ACCGAGCTTA	ACTCTGGGGC TGTAGCACCA
*OCL*	AGCAGGAGGGCAATAAGGT	TTTGTAGGCGGTCTTCAAGC
*OPN*	ACACTTTCACTCCAATCGTCC	TGCCCTTTCCGTTGTTGTCC
*GAPDH*	ACCACAGTCCATGCCATCAC	TCCACCACCCTGTTGCTGTA
*HPRT1*	TGCTCGAGATGTCATGAAGG	AGAGGTCCTTTTCACCAGCA
